# Oral appliance therapy versus nasal continuous positive airway pressure in obstructive sleep apnea: a randomized, placebo-controlled trial on psychological distress

**DOI:** 10.1007/s00784-016-2045-3

**Published:** 2017-01-12

**Authors:** Ghizlane Aarab, Maria Nikolopoulou, Jari Ahlberg, Martijn W. Heymans, Hans L. Hamburger, Jan de Lange, Frank Lobbezoo

**Affiliations:** 1 Department of Oral Kinesiology, Academic Centre for Dentistry Amsterdam (ACTA), University of Amsterdam and VU University Amsterdam, MOVE Research Institute Amsterdam, Gustav Mahlerlaan 3004, 1081 LA, Amsterdam, The Netherlands; 2 Department of Oral and Maxillofacial Diseases, University of Helsinki, Helsinki, Finland; 3 Department of Epidemiology and Biostatistics, VU University Medical Center, Amsterdam, The Netherlands; 4 Department of Clinical Neurophysiology and Center for Sleep-Wake Disorders, Slotervaart Medical Center, Amsterdam, The Netherlands; 5 Department of Oral and Maxillofacial Surgery of the Academic Medical Centre of the University of Amsterdam and Academic Centre for Dentistry Amsterdam (ACTA), Amsterdam, The Netherlands

**Keywords:** Psychology, Psychiatry, Obstructive sleep apnea, Mandibular, Repositioning, Splint

## Abstract

**Objectives:**

The aim of this randomized placebo-controlled trail was to compare the effects of an objectively titrated mandibular advancement device (MAD) with those of nasal continuous positive airway pressure (nCPAP) and an intraoral placebo device on symptoms of psychological distress in OSA patients.

**Materials and methods:**

In a parallel design, 64 mild/moderate OSA patients (52.0 ± 9.6 years) were randomly assigned to an objectively titrated MAD, nCPAP, or an intraoral placebo appliance. All patients filled out the Symptom Checklist-90-Revised twice: one before treatment and one after 6 months of treatment. The Symptom Checklist-90-Revised is a multidimensional symptom inventory designed to measure symptomatic psychological distress over the past week. Linear mixed model analyses were performed to study differences between the therapy groups for the different dimensions of the Symptom Checklist-90-Revised over time.

**Results:**

The MAD group showed significant improvements over time in the dimensions “somatization,” “insufficiency of thinking and acting,” “agoraphobia,” “anxiety,” “sleeping problems,” and “global severity index” (*F* = 4.14–16.73, *P* = 0.048–0.000). These improvements in symptoms of psychological distress were, however, not significantly different from those observed in the nCPAP and placebo groups (*P* = 0.374–0.953).

**Conclusion:**

There is no significant difference between MAD, nCPAP, and an intraoral placebo appliance in their beneficial effects on symptoms of psychological distress.

**Clinical relevance:**

The improvement in psychological distress symptoms in mild/moderate OSA patients under MAD or nCPAP treatment may be explained by a placebo effect.

## Introduction

Obstructive sleep apnea (OSA) is characterized by recurrent obstruction of the upper airway, often resulting in oxygen desaturation and arousal from sleep [1]. Excessive daytime sleepiness, snoring, and reduction in cognitive functions are common symptoms of this condition [1]. OSA patients may also report symptoms of psychological distress, such as depression and anxiety [2]. Beebe and Gozal [3] suggested that both intermittent hypoxia and sleep disruption induce dysfunction of the prefrontal regions of the brain cortex, which may predispose to psychological distress.

Although continuous positive airway pressure (CPAP) has been proposed as the most effective treatment for severe OSA patients, nowadays mandibular advancement devices (MADs) are considered as a primary treatment option in mild and moderate OSA patients and in patients who do not tolerate CPAP [4]. The rationale behind the efficacy of MADs is that advancement of the mandible and tongue improves upper airway patency during sleep by enlarging the upper airway and by decreasing upper airway collapsibility [5, 6].

Barnes et al. [7] compared the effects of MAD treatment with CPAP on mood disorders and depression in a randomized placebo-controlled crossover trial and found no significant differences between these two therapies in their improvement of these disorders. Similar results were found by Engleman et al. [8] in a randomized crossover trial in which the effects of CPAP and MAD treatment on anxiety and depression symptoms were compared. To our best knowledge, no randomized placebo-controlled trials have been performed comparing the effects of an objectively titrated MAD and CPAP on symptoms of psychological distress. To enable an unbiased comparison between those treatment modalities, both treatments should be titrated objectively. Further, the crossover design of previous studies may have a risk of carry-over effects. The primary aim of this randomized placebo-controlled trial was, therefore, to compare the effects of an objectively titrated MAD with those of nasal CPAP (nCPAP) and an intraoral placebo appliance on symptoms of psychological distress in a parallel design. The hypothesis was that there is no significant difference between objectively titrated MAD and nCPAP therapies in improving psychological distress symptoms in mild/moderate OSA patients. To control for possible placebo effects, an intraoral placebo device served as a passive control condition for both active treatment modalities. It was hypothesized that the intraoral placebo appliance would not significantly improve psychological distress symptoms in mild/moderate OSA patients. Following the hypothesis of Beebe and Gozal [3], we also hypothesized that a significant correlation between the amount of psychological distress and the apnea-hypopnea index (AHI) values in the three therapy groups would occur. Therefore, the secondary aims of this trial were (1) to determine the relation between the amount of psychological distress and the AHI values at baseline in the three therapy groups and (2) to determine the relation between the amount of psychological distress at baseline and the change of AHI over time in the three therapy groups.

## Patients and methods

### Setting and participants

This study is part of a randomized controlled trial (RCT), in which three therapy groups (viz., MAD, nCPAP, and placebo) were compared [9]. Eligible OSA patients, living in the greater Amsterdam area, were referred to the Slotervaart Medical Center by their family physician. All patients underwent a thorough medical examination, including a full polysomnographic (PSG) recording, at the Departments of Neurology, Pulmonary Medicine, and ENT, as well as a thorough dental examination at the Department of Oral Kinesiology of the Academic Centre for Dentistry Amsterdam (ACTA). The OSA patients were invited for participation in the study when they fulfilled the following inclusion criteria: age >18 years, an apnea-hypopnea index (AHI) between 5 and 45 events per hour, and an Epworth sleepiness score (ESS) ≥10 or at least two of the symptoms suggested by the American Academy of Sleep Medicine Task Force, e.g., unrefreshing sleep and daytime fatigue [1, 10]. The medical and dental exclusion criteria are shown in Table 1 [9]. Exclusion of temporomandibular disorders was based on a functional examination of the masticatory system [11, 12].

**Table 1:** Number of patients excluded based on the medical and dental exclusion criteria used in this study [9]

Exclusion criteria	Number of patients excluded
Medical	
Respiratory/sleep disorder other than OSA	23
Body mass index >40	3
Medication usage that could influence respiration or sleep	2
Periodic limb movement disorder	21
Previous treatment with CPAP or MAD	–
Reversible morphological upper airway abnormalities (e.g., enlarged tonsils)	17
Other medical conditions (e.g., psychiatric disorder)	7
Dental	
Temporomandibular disorders	–
Untreated periodontal problems	1
Dental pain	–
Lack of retention possibilities for an oral appliance	28

The baseline characteristics of the patients at the time of therapy allocation are presented in Table 2. This study was approved by the Slotervaart Medical Center’s Ethics Committee (# U/1731/0326, U/2679/0326). Written informed consent was obtained from all participants. This study has been registered at www.clinicaltrials.gov (# NCT00950495).

**Table 2:** Patient characteristics (mean ± SD) at baseline of the mandibular advancement device (MAD) group, nasal continuous positive airway pressure (nCPAP) group, placebo group, and drop-outs group and the normal values in the healthy Dutch population for the various dimensions of the Symptom Checklist-90-Revised (SCL-90-R)

	MAD (*n* = 21)	nCPAP (*n* = 22)	Placebo (*n* = 21)	Drop-outs (*n* = 7)	Normal values of Dutch healthy population (*n* = 1004)
Age (years)	50.4 ± 8.9	54.0 ± 10.1	51.3 ± 9.6	49.3 ± 7.3	–
Number of man/woman	17/ 4	15/ 7	15/ 6	5/ 2	–
Apnea-hypopnea index	21.4 ± 11.0	20.1 ± 9.0	19.5 ± 8.4	14.8 ± 3.8	–
Epworth sleepiness score	12.0 ± 5.7	10.7 ± 4.4	10.8 ± 4.0	13.7 ± 1.9	–
Body mass index (kg/m^2^)^a^	27.1 ± 3.2	30.7 ± 3.7	31.1 ± 4.7	27.8 ± 4.1	
SCL-90-R					
Somatization	22.0 ± 10.3	24.6 ± 11.9	21.9 ± 10.8	24.3 ± 10.3	16.7 ± 5.3***
Insufficiency of thinking and acting	18.3 ± 7.6	18.7 ± 9.6	19.9 ± 9.5	19.0 ± 6.7	12.6 ± 4.3***
Interpersonal sensitivity	27.6 ± 10.4	26.9 ± 14.5	28.5 ± 17.7	26.3 ± 8.5	24.1 ± 7.6
Depression	26.3 ± 11.8	28.5 ± 15.3	30.5 ± 17.5	30.3 ± 15.8	21.6 ± 7.6***
Anxiety	14.9 ± 6.5	16.9 ± 9.6	15.6 ± 9.7	14.8 ± 5.6	12.8 ± 4.4*
Hostility	8.8 ± 3.7	9.2 ± 3.8	8.6 ± 4.6	8.8 ± 1.9	7.2 ± 2.1**
Agoraphobia	8.7 ± 3.0	9.3 ± 5.1	9.2 ± 6.7	7.5 ± 1.0	7.9 ± 2.3
Sleeping problems	7.6 ± 3.6	7.2 ± 3.8	8.4 ± 4.3	10.4 ± 4.6	4.5 ± 2.2***
Global severity index	149.3 ± 60.3	144.9 ± 68.1	162.0 ± 90.7	118.0 ± 38.2	118.3 ± 32.4*

*P* values as result of the one-sample *t* tests comparing the three therapy groups and the normal values in Dutch healthy population for the various dimensions of the SCL-90-R: ***P < 0.001; **P < 0.01; *P < 0.05

^a^MAD patients had a significantly lower BMI than placebo and nCPAP patients (*P* = 0.002 and 0.006, respectively)

### Randomization and interventions

At the start of this RCT, consenting patients were allocated to the interventions using block randomization. The allocation sequence was automatically generated and concealed by an independent co-worker. Three types of interventions were used in this parallel-group study. First, an individually fabricated MAD with an adjustable mandibular protrusion position at a constant vertical dimension was used [13, 14]. Second, nCPAP of the REMstar Pro system was used (Respironics, Herrsching, Germany). Third, a thin (<1 mm), hard acrylic-resin palatal splint with only a partial palatal coverage was used as a placebo [15].

Both MAD and nCPAP were titrated before the start of the treatment [9]. For the titration of the MAD, four ambulatory polysomnographic (PSG) recordings were performed at regular time intervals of approx. 3 weeks. The total titration period was approx. 10 weeks. The most effective protrusion position of the MAD (i.e., the mandibular position that yielded the lowest AHI value) was chosen from among four randomly offered positions (viz., 0, 25, 50, and 75% of the maximum protrusion). The MAD was set at 25% of the maximum protrusion in 1 patient, at 50% in 7 patients, and at 75% in 12 patients [9]. For the placebo group, four ambulatory PSG recordings were performed at regular time intervals similar to the MAD group [16]. The titration of nCPAP was performed during a PSG recording at the Slotervaart Medical Center. The pressure was increased in steps of 1 cm H_2_O/hour, until the AHI and respiration-related arousals were reduced to ≤5/hour, and snoring was minimized. The average value of the pressure was 7.3 (SD, 1.9; range, 4–11) cm H_2_O [9].

### Procedure

During the titration period of approx. 10 weeks, all the patients visited ACTA four times at regular intervals, during which the BMI (kg/m^2^) was determined and the Epworth sleepiness scale (ESS) [10] was completed. The participants were also interviewed (1) about their compliance (% of nights per week of wearing), (2) about possible side effects (nature and number; determined in an open question) of the MAD during the study period, and (3) about the change (increased, unchanged, or decreased) in snoring intensity, based on information they obtained from their bed partner. These outcomes have been described in detail in Aarab et al. [13] and Aarab et al. [9].

From all the patients, two PSG recordings were obtained in the sleep laboratory of the Slotervaart Medical Center: the first one before treatment and the second one after 6 ± 2 months (mean ± SD) for the therapy evaluation. The outcomes of these PSG recordings are also described in detail in Aarab et al. [9].

All patients filled out the Dutch version of the Symptom Checklist-90-Revised (SCL-90-R) twice: the first one before treatment and the second one at therapy evaluation. The SCL-90-R is a multidimensional symptom inventory designed to measure symptomatic psychological distress over the past week (e.g., depression, anxiety, and somatization). Its reliability and validity proved to be good for both the original and the Dutch version [17, 18]. Moreover, norm scores are available for the Dutch general population [17].

### Data analysis

The patient characteristics of the three therapy groups at baseline, including the different dimensions of the SCL-90-R, were compared using one-way analyses of variance, followed by least-significant difference (LSD) pair-wise comparisons. One-way analyses of variance were also used to detect differences in compliance between the three therapy groups [9]. For the different dimensions of the SCL-90-R, one-sample *t* tests were used to analyze differences between outcomes related to the therapy groups and the normal values of the Dutch population, and model assumptions were checked. For both the per-protocol analysis and the intention-to-treat analysis, linear mixed models were used to study the differences between the groups for the different dimensions of the SCL-90-R over time. In these models, the treatment group variable was introduced as a dummy variable with the MAD group as reference group. The difference between treatment groups over time was studied by an interaction term of treatment times the time variable. Pearson’s correlation was used to test the relation between AHI values and the different dimensions of the SCL-90-R.

All statistical tests were performed with the SPSS 21.0 (SPSS Inc., Chicago, IL) and SAS 9.3 (Statistical Analysis System, SAS Institute Inc., Cary, NC, USA) software packages.

## Results

**Fig. 1:**
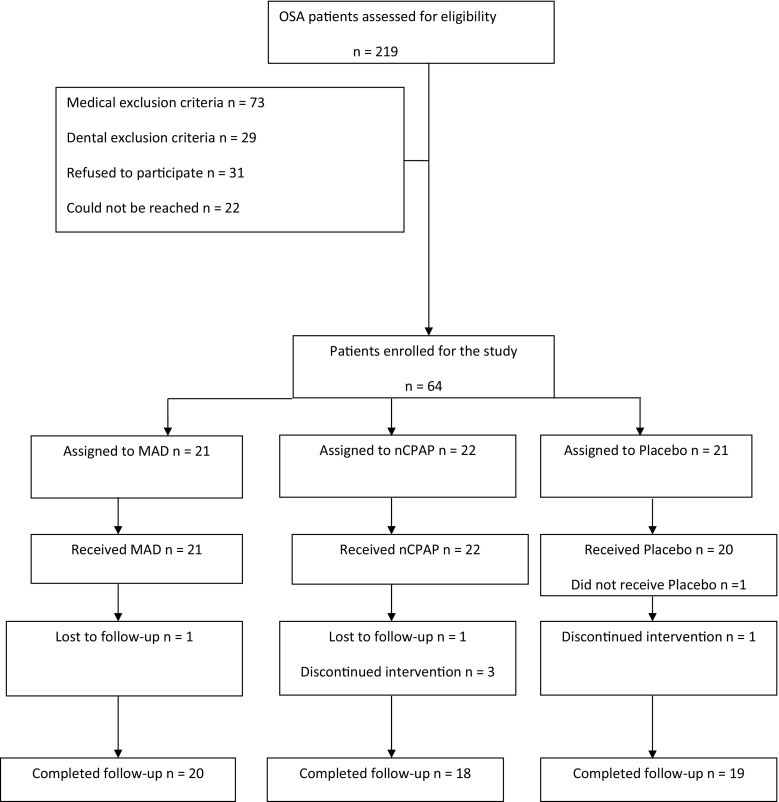
Flow-chart of the patients through each stage of the trial. *MAD* mandibular advancement device, *nCPAP* nasal continuous positive airway pressure

A total of 64 patients were enrolled in the study and were randomized at the start of the RCT as shown in Fig. 1 [9]. Three patients in the nCPAP group terminated the treatment before evaluation, because they experienced more side effects than benefits out of their treatment. One patient in the placebo group terminated the treatment, because of private reasons unrelated to the study. Another patient in the placebo group did not receive the placebo treatment, because of an urgent medical condition that occurred after the allocation. Two other patients, one in the nCPAP group and another in the MAD group, could not be reached after the random allocation and could thus not be evaluated. Hence, 57 patients completed the entire study protocol.

The patient characteristics at baseline are presented in Table 2. BMI was the only baseline characteristic that differed between the three therapy groups (*F* = 5.170; *P* = 0.008). LSD analyses revealed that the MAD group had a significantly lower BMI than the placebo and nCPAP groups (*P* = 0.002 and 0.006, respectively) [9]. The mean (±SD) baseline values of the different dimensions of the SCL-90-R of the three therapy groups, of the drop-outs, and of the normal values of the Dutch healthy population are also shown in Table 2. The baseline values of the different dimensions did not differ significantly between the three therapy groups (*P* = 0.305–0.987; Table 2). Further, the baseline values of the SCL-90-R of the drop-outs were not different from those of the therapy groups either (*P* = 0.348–0.997). The three groups showed higher average values of psychological distress at baseline than the reported normal values for the Dutch population in the dimensions “somatization,” “insufficiency of thinking and acting,” “anxiety,” “hostility,” “depression,” “sleeping problems,” and “global severity index” (*T* = 6357–2.566; *P* = 0.000–0.013; Table 2).

The mean (±SD) baseline values of the different dimensions of the SCL-90-R of the three therapy groups who completed the entire study protocol as well as the changes in these variables from baseline to therapy evaluation are shown in Table 3. As a result of missing values in the different dimensions of the SCL-90-R per therapy group, the number of observations used in the per-protocol analyses varied per dimension (see Table 3). The MAD group showed significant improvements over time in the dimensions “somatization,” “insufficiency of thinking and acting,” “agoraphobia,” “anxiety,” “sleeping problems,” and the “global severity index” (*F* = 4.01–15.47, *P* = 0.048–0.000, Table 3). These improvements in symptoms were, however, not significantly different from the improvements in symptoms observed in the nCPAP and placebo groups (*P* = 0.374–0.953). The intention-to-treat analysis showed similar results as the per-protocol analyses: the MAD group showed significant improvements over time in the dimensions “somatization,” “insufficiency of thinking and acting,” “agoraphobia,” “anxiety,” “sleeping problems,” and the “global severity index” as well (*F* = 4.01–16.34, *P* = 0.025–0.000), while these improvements were not significantly different from those observed in the nCPAP and placebo groups (*P* = 0.175–0.950).

**Table 3:** The mean (±SD) baseline and therapy evaluation values of the different dimensions of the Symptom Checklist-90-Revised (SCL-90-R) of the mandibular advancement device (MAD) group, nasal continuous positive airway pressure (nCPAP) group, and placebo group in the per-protocol analyses

Dimension (number of observations used)	MAD (*n* = 20)		P*	nCPAP (*n* = 18)		Placebo (*n* = 19)		P*
	Baseline	Therapy		Baseline	Therapy	Baseline	Therapy	
Somatization (*n* = 107)	22.0 ± 10.3	17.7 ± 5.5	0.000**	24.6 ± 11.9	21.3 ± 11.5	21.9 ± 10.8	17.9 ± 7.9	0.374
Insufficiency of thinking and acting* (*n* = 104)	18.3 ± 7.6	15.8 ± 5.7	0.003**	18.7 ± 9.6	17.7 ± 10.0	19.9 ± 9.5	16.3 ± 8.1	0.646
Interpersonal sensitivity (*n* = 102)	27.6 ± 10.4	25.3 ± 8.4	0.206	26.9 ± 14.5	27.3 ± 14.8	28.5 ± 17.7	25.4 ± 15.4	0.953
Depression (*n* = 96)	26.3 ± 11.8	24.0 ± 7.1	0.056	28.5 ± 15.3	25.4 ± 16.4	30.5 ± 17.5	23.8 ± 6.5	0.445
Anxiety (*n* = 104)	14.9 ± 6.5	12.9 ± 4.2	0.033**	16.9 ± 9.6	15.2 ± 9.1	15.6 ± 9.7	14.1 ± 6.8	0.573
Hostility (*n* = 106)	8.8 ± 3.7	8.7 ± 3.3	0.437	9.2 ± 3.8	8.9 ± 3.8	8.6 ± 4.6	7.5 ± 2.6	0.521
Agoraphobia (*n* = 103)	8.7 ± 3.0	7.7 ± 1.1	0.028**	9.3 ± 5.1	7.8 ± 1.9	9.2 ± 6.7	8.9 ± 5.5	0.479
Sleeping problems (*n* = 106)	7.6 ± 3.6	6.3 ± 3.0	0.048**	7.2 ± 3.8	6.1 ± 2.5	8.4 ± 4.3	7.4 ± 3.3	0.792
Global severity index (*n* = 85)	149.3 ± 60.3	132.9 ± 38.2	0.018**	144.9 ± 68.1	137.7 ± 66.2	162.0 ± 90.7	124.1 ± 24.0	0.387

*P value as result of the linear mixed model analyses for the time effect within the MAD group

**Statistically significant at the 0.05 probability level

***P value as result of the linear mixed model analyses comparing the three groups over time

The MAD group had used their appliance 90.6% (SD, 13.3) of the nights; the nCPAP group 82.9% (SD, 27.2) of the nights; and the placebo group 93.9% (SD, 15.7) of the nights. No significant group differences in compliance were found (*F* = 1.518, *P* = 0.228) [9].

**Fig. 2:**
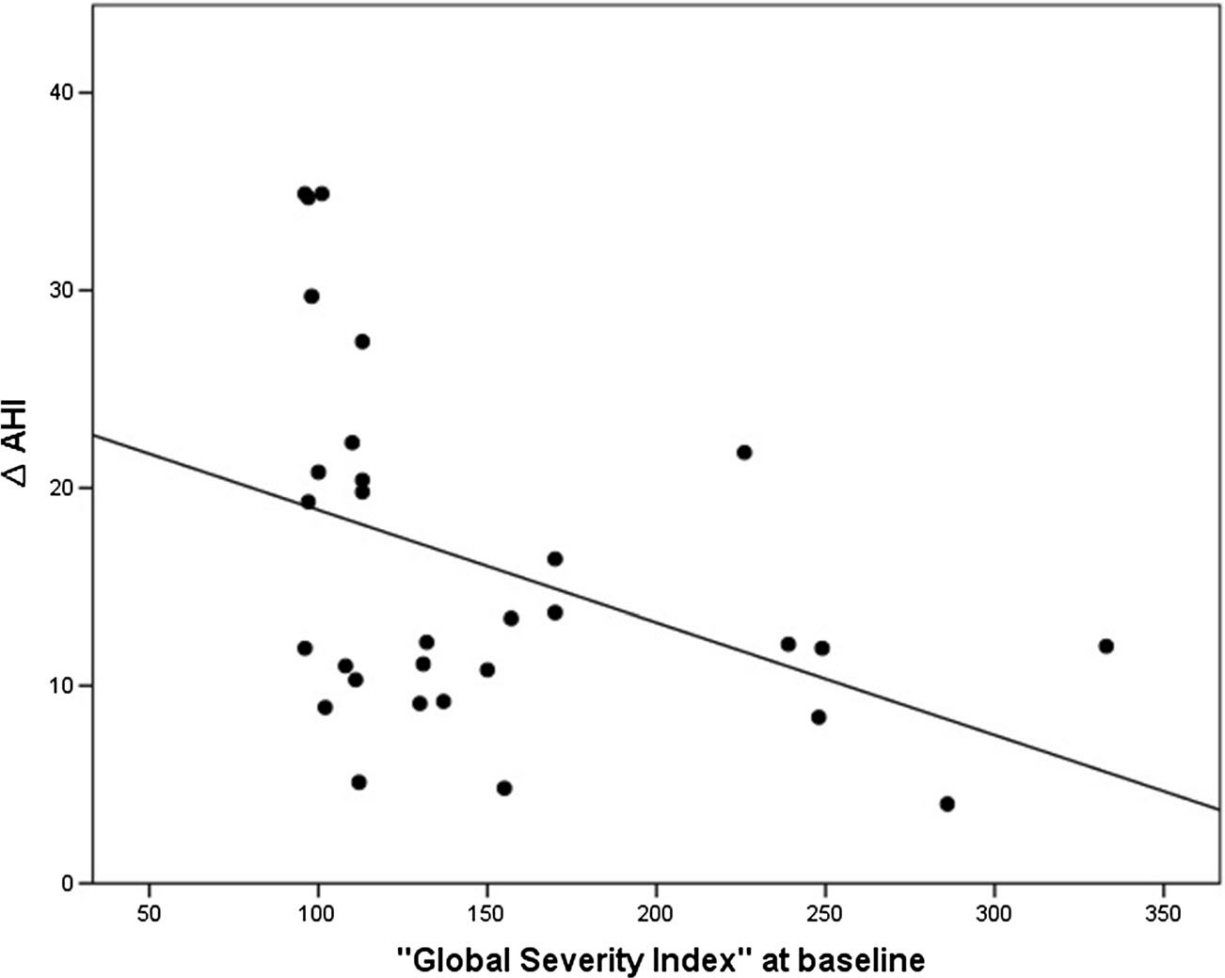
Scatterplot of the correlation between the baseline values of the “global severity index” and the changes in AHI values between baseline and therapy evaluation (∆AHI) in both the MAD and nCPAP groups

There was no significant correlation between the baseline AHI value and the baseline values of the different dimensions of the SCL-90-R in the three groups (*P* = 0.121–0.888). A significant correlation was found between the baseline values of the “global severity index” and the changes in AHI values (∆AHI) between baseline and therapy evaluation in both the MAD and nCPAP group (*P* = 0.025). Patients with higher values of the “global severity index” at baseline showed less reduction in the AHI than patients with lower values of this index at baseline (Fig. 2). In the placebo group, there was no significant correlation between “∆AHI” and the baseline values of “global severity index” (*P* = 0.615).

## Discussion

Both MAD and nCPAP showed significant improvements of symptoms of psychological distress after 6 months of treatment. However, these significant improvements were not different from those observed in the placebo group.

In randomized clinical trials, there are often problems of noncompliance, where the patient does not adhere to the assigned treatment or does not complete questionnaires as we also observed in this study. Typically, this leads to estimates that can potentially be biased when the probability of a missing value is related to the characteristics of the patients. Further, missing data can also lead to a reduction of statistical power [19]. To overcome this problem, linear mixed model analysis can be used. The major strengths of mixed models are their ability to accommodate missing data points often encountered in longitudinal datasets and to generate valid study results [20]. Therefore, we used linear mixed model analyses in this study.

The population in the present study showed higher average values of psychological distress at baseline than the reported normal values for the Dutch population. The relationship between OSA and psychiatric disorders, especially depression, has already been studied for decades [21]. Pillar and Lavie [22] reported in their male population that neither the presence nor the severity of OSA was associated with depression or anxiety. On the other hand, recent evidence has confirmed important connections between OSA and psychiatric disorders. Psychiatric co-morbidity in OSA patients was examined in a large retrospective chart review of more than 100,000 veterans. A significantly higher prevalence of numerous psychiatric disorders, including depression and anxiety, was found in OSA patients as compared to non-OSA patients [23]. Others reported depression symptoms in 17–41% of OSA patients [24, 25]. Harris et al. [2] suggested that direct treatment of depression in OSA patients might improve acceptance of therapy, reduce sleepiness and fatigue, and improve quality of life, but that intervention trials are needed to answer this question. Although the causal relationship between symptoms of psychological distress and OSA has not been determined yet, a higher prevalence of these symptoms in OSA patient seems to be a consistent finding, which corresponds with our results.

All patients who completed the trial showed relatively high compliance rates of approx. 90% (i.e., the percentage of nights per week usage). This relatively high compliance may be explained by the fact that during the study period the patients frequently visited ACTA to be interviewed about the frequency of wearing. This regular contact with the examiner could have motivated the patients to use their appliances frequently [9]. Although self-reported compliance has been suggested to overestimate the actual use of MADs, covert compliance monitoring has shown excellent agreement between subjective and objective compliance [26, 27].

The MAD effects on the OSA condition have been compared with those of CPAP in several randomized clinical trials [4]. Although in most previous crossover studies MADs were considered less effective in reducing the AHI value than CPAP in mild-to-moderate OSA patients, similar improvements in subjective outcomes, such as excessive daytime sleepiness and quality of life, were found [8, 28–30]. Further, it should be noted that these studies also indicated that, in general, patients find MADs as a more acceptable treatment compared to CPAP. In recent RCTs with a parallel design, no significant differences between MAD and nCPAP were reported in the subjective outcomes [9, 31]. Aarab et al. [9] found no significant difference in efficacy between MAD and CPAP in mild-to-moderate cases. Although Doff et al. [31] showed that CPAP was more effective in lowering the AHI than MAD in a group of mild to severe OSA patients, they found no significant differences between both treatments in the proportions of successful treatments. A recent meta-analysis showed that CPAP is more effective in lowering AHI than MAD in moderate-to-severe OSA patients; however, the superiority of CPAP over MAD is hypothesized to be less in mild cases [32]. Further, a recent crossover study by Phillips et al. [33] showed that important health outcomes were similar after 1 month of optimal MAD and CPAP treatment in patients with moderate-to-severe OSA. Thus, the outcomes of our study are in line with previous findings wherein both MAD and nCPAP show comparable treatment results in a group of mild-to-moderate OSA patients.

Beebe and Gozal [3] suggested that both intermittent hypoxia and sleep disruption induce dysfunction of the prefrontal regions of the brain cortex, which may predispose to mood disorders. Following this hypothesis, at baseline, we suspected a significant correlation between the amount of psychological distress and the AHI values. However, we did not find this correlation. On the other hand, OSA patients with higher values of the psychological distress at baseline showed less reduction in the AHI than patients with lower values of this index at baseline. The nature of this association is unclear, but this finding suggests that the level of psychological distress at the start of the treatment may play a significant role in the treatment outcome.

In this study, the significant improvements in symptoms of psychological distress in the MAD and nCPAP groups were not better than those observed in the placebo group. This is in line with our previous findings wherein we reported significant improvements in the Epworth sleepiness scale (ESS) and the Short-Form General Health Survey (SF-36) in all three groups without any differences in effects between the three therapy groups [9]. These placebo effects on excessive daytime sleepiness were also shown in a recent study, in which the effects of MAD were compared with an intraoral placebo splint in mild-to-moderate OSA patients [34]. Power calculation was performed for the primary outcome variable of this randomized placebo-controlled trial, viz., the AHI [9]. No power calculations were performed for the secondary outcome variables (viz., ESS, SF-36, and SCL-90-R). Therefore, our sample size per therapy group may have not been sufficient to find a significant difference between the therapy groups in the change of the different dimensions of SCL-90-R. However, our findings correspond with many previous, well-designed studies [7, 28, 35, 36] in which it was also reported that most of their OSA patients obtained a significant benefit in neuropsychological function and mood from their placebo treatment compared to MAD and CPAP treatments. These observed improvements in symptoms of psychological distress may be due to extensive attention given to the patients during the entire protocol, to a change in lifestyle as a result of the information given to the patients at baseline, and/or to a placebo response. Further, the high initial values of the SLC-90-R scores at baseline result in a higher possibility of decreases in these scores over time. The tendency of high values to return towards an individual’s more typical average state is known as “regression to the mean.” Stepnowsky et al. [37] reported in a recent study that baseline emotional distress predicted the drop in AHI in response to placebo treatment. Highly distressed patients showed a greater placebo response with a 34% drop in AHI. Although we could not confirm this in our study, all these findings together support the importance of including a placebo treatment in a randomized controlled trial design to determine unbiased treatment effects.

Within the limits of this study, it can be concluded that there is no significant difference between MAD, nCPAP, and an intraoral placebo appliance in their beneficial effects on symptoms of psychological distress in mild-to-moderate OSA patients. Further, this study suggests that the level of psychological distress at the start of the treatment may play a significant role in the treatment outcome of MAD and nCPAP in a group of mild-to-moderate OSA patients.
